# Myotendinous Thermoregulation in National Level Sprinters after a Unilateral Fatigue Acute Bout—A Descriptive Study

**DOI:** 10.3390/s23239330

**Published:** 2023-11-22

**Authors:** Alessio Cabizosu, Cristian Marín-Pagán, Antonio Martínez-Serrano, Pedro E. Alcaraz, Francisco Javier Martínez-Noguera

**Affiliations:** 1 THERMHESC Group, Chair of Molina Ribera Hospital, C. Asociación, S/N, 30500 Molina de Segura, Spain; acabizosu@ucam.edu; 2 Research Center for High Performance Sport, Catholic University of Murcia (UCAM), Campus de los Jerónimos, Nº 135, 30107 Murcia, Spain; amartinez30@ucam.edu (A.M.-S.); palcaraz@ucam.edu (P.E.A.); fjmartinez3@ucam.edu (F.J.M.-N.)

**Keywords:** thermography, tendons, quadriceps muscle, body temperature regulation

## Abstract

In the last decade there has been a growing interest in infrared thermography in the field of sports medicine in order to elucidate the mechanisms of thermoregulation. The aim of this study was to describe bilateral variations in skin temperature of the anterior thigh and patellar tendon in healthy athletes and to provide a model of baseline tendon and muscle thermoregulation in healthy sprinters following a unilateral isokinetic fatigue protocol. Fifteen healthy national-level sprinters (eleven men and four women), with at least 3 years of athletic training experience of 10–12 h/week and competing in national-level competitions, underwent unilateral isokinetic force testing and electrostimulation in which their body temperature was measured before, during, and after the protocol using an infrared thermographic camera. ANOVA detected a significant difference in the time × side interaction for patellar temperature changes (*p* ≤ 0.001) and a significant difference in the time/side interaction for quadriceps temperature changes (*p* ≤ 0.001). The thermal challenge produces homogeneous changes evident in quadriceps areas, but not homogeneous in tendon areas. These data show that metabolic and blood flow changes may depend on the physical and mechanical properties of each tissue. Future research could be conducted to evaluate the predictive value of neuromuscular fatigue in the patellar tendon and quadriceps after exercise in order to optimize post-exercise recovery strategies.

## 1. Introduction

Since it has been observed that cutaneous infrared radiation changes may be evidence of acute physiological responses, thermography has been the subject of numerous studies in sports medicine, due to the possibility of obtaining immediate data about the functional state of the studied structures is a great advantage over other techniques [1,2].

Because it is inexpensive, non-invasive, and free of contraindication tools [3,4], in order to clarify the thermoregulatory processes and the physiological and metabolic responses to exercise, different studies have been carried out in many disciplines using infrared thermography [5,6,7].

According to the microscopic concept of temperature, it is known that this measure corresponds to the average value of the kinetic energy of all the particles that constitute matter [8]. It is known that substances are composed of numerous particles that have a disordered movement, and the temperature indicates the degree of molecular agitation. So, the greater the molecular agitation, the greater the kinetic energy, and this is reflected in a higher temperature [9]. If we consider that, in humans, there are several conditions that can generate an increase or decrease in cellular activity, it is easy to understand that thermography can be a very useful tool for the assessment of the physiological state of the human being [10].

However, it should be noted that there are many other biomedical fields, in addition to sports medicine, in which this technique is being implemented exponentially. In health sciences for example the use of this technique has been extended to different medical areas such as neurology [11], cardiology [12], endocrinology [13], and dermatology [14].

Although it is currently considered mainly as a complementary diagnostic tool, there are already numerous studies that attempt to classify certain diseases on the basis of the differences in thermographic patterns obtained in an attempt to validate the thermographic tool as a reliable diagnostic or monitoring technique [15,16].

In fact, associating, for example, the use of this technique to the Doppler signal, recent studies have demonstrated its great usefulness in the analysis of patellar tendinitis in young athletes [17], or studies comparing CT images with thermographic images have allowed important advances in the field of rare neurodegenerative diseases [3], however, it should be noted that more and more research is demonstrating good inter- and intra-examiner reliability [18] and the diagnostic validity of this tool [19].

The fact that this diagnostic technique is easily transportable, due to its weight and size of approximately 1 kg (2.2 lbs) and that it is also a quick and easy-to-use technique, has encouraged its use progressively in sports medicine, being currently a very reliable and objective technique of metabolic response, before, during, and immediately after competitions.

Initially, sports such as soccer and basketball have been the subject of numerous studies [20,21,22,23]. However, there is currently a growing increase in the literature on running sports due to the interest generated by the different thermoregulatory responses under prolonged muscular stress [24].

It is known that high-performance training generates important physiological changes at a systemic level, so some studies carried out on runners analyze the role of thermography in relation to sports performance and muscular response [25,26,27] so that assessing the thermoregulatory response before, during, and after exercise is essential to more accurately establish workloads in individual athletes. To this end, the analysis of differences or associations between skin temperature and musculoskeletal response is currently a priority in the scientific field related to competitive running sports, with bilateral metabolic responses being analyzed mostly in the days following physical exercise, but not exhaustively in the short term [28,29].

In this regard, it should be noted that despite the effort being made to generate new knowledge in the field of thermography, either in mathematical analysis methods of thermal patterns [30] or in the automation and standardization of the diagnostic process with infrared biomedical images [31], there are still many aspects that need to be investigated and clarified.

Due to the difficulty of reproducing measurement protocols and environmental and metabolic conditions [32], studies of analysis and interpretations of normal thermographic data are quantitatively scarce, so research is currently focused on analyzing thermographic values after immediate exertion or 24–48 h after exertion, in the unilaterality/bilaterality relationship, especially in healthy runners [28,29].

In this sense, previous studies have shown that depending on the exercise performed, the exercise intensity, and the relationship with certain biochemical markers related to muscle damage, the skin responds by altering its basal thermal state [33,34,35]. Therefore, the technique of skin surface temperature assessment can be considered as a thermoregulatory indicator of acute metabolic stress and fatigue at the muscle level.

Therefore, the aim of this study was to describe bilateral variations in skin temperature of the anterior thigh and patellar tendon in healthy athletes and to provide a model of baseline tendon and muscle thermoregulation in healthy sprinters following, in situ, a unilateral isokinetic fatigue protocol.

## 2. Material and Method

### 2.1. Study Design

This descriptive study consisted of 2 laboratory visits. Visit 1 consisted of a medical examination to determine health status and an isokinetic familiarization session. In visit 2, athletes performed the protocol described in Section 2.3.2, where patellar skin surface temperature was measured at baseline (B), after warm-up (W), after 1st electrostimulation (1st ELEC), after isokinetic (ISO), and after 2nd electrostimulation (2nd ELEC). In the quadriceps, skin surface temperature was measured at BA after 1st ELEC, after ISO, and after 2nd ELEC. In addition, before each testing session (visit 2), the nutritionist prescribed a standardized breakfast 2 h before the rectangular test, which consisted of 1.30 g/BW carbohydrate, 0.43 g/BW protein, and 0.57 g/BW fat.

### 2.2. Participants

Fifteen healthy national-level sprinters were recruited (eleven men and four women). All subjects had to meet the following inclusion criteria: (a) age 18–30 years; (b) BMI 19.0–25.5 kg·m^2^; and (c) at least 3 years of athletic training experience of 10–12 h/week and compete in national level competitions. Subjects were excluded if they: (a) had any metabolic or cardiovascular pathology or abnormality; (b) smoked or drank regularly; (c) took any supplements or medication in the previous 2 weeks; (d) had suffered any injury in the last 6 months; (e) had received physiotherapy 24 h before the thermographic measurements; (f) had a pathological or metabolic disease that could alter the thermographic results; and (g) were taking any supplement or medication that could affect thermoregulation. The study was conducted in accordance with the Declaration of Helsinki for Human Research [36] and was approved by the Ethics Committee of the Catholic University of Murcia (CE102201). All participants were informed of the study procedures and signed informed consent forms; in the case of underage athletes, their relatives were informed and signed the informed consent forms.

### 2.3. Assessments

#### 2.3.1. Medical Exam and Familiarization Session

A medical examination included a health history, a resting electrocardiogram, and a cardiorespiratory examination (auscultation, blood pressure, etc.), and confirmed that the volunteer was healthy to participate in the study.

In the familiarization session, participants were placed in the assessment position (isokinetic chair device) and electrostimulations were applied to familiarize them with the stimulus.

#### 2.3.2. Thermography Protocol and Muscular Stress Test

We used a Flir E75 camera (Wilsonville, OR, EE.UU) with an infrared resolution of 320 × 240 pixels and thermal sensitivity of <0.04 °C. This infrared camera offers wide measuring ranges (from −20 to 120 °C, from 0 to 650 °C, and from 300 °C to 1000 °C), with the range from −20 to 120 °C having been used in this study. The emissivity was set to 0.98 according to the bibliographic indications regarding the skin study [37,38]. The spectral range was 7.4–14.0 micrometer (μm), and the detector type was a non-refrigerated microbolometer of 17 μm. Measurement accuracy and frame rate were ±2 °C or ±2% of the reading, the ambient temperature was from 15 to 35 °C, and the object temperature was higher than 0 °C and 30 Hz. The minimum focal length was 0.5 m.

The athletes were positioned with shorts on a 1.5 m thick cotton pad to avoid direct contact with the floor and, thus, generate temperature changes in the lower limbs. To allow the correct configuration of the machine in the room, the principal investigator turned on the machine one hour before the first recording was made.

The thermographic protocol was carried out in different phases following the TISSEM protocol [38]. Data were collected by the sex, height, weight, and BMI of the participants. In addition, information was collected on the last physiotherapy and training sessions performed. High-intensity sessions could not have been performed 48 h before the study to avoid alterations in the metabolic responses of the muscular system. Participants were informed beforehand that they could not sunbathe, use chemicals on the skin, use drugs, or drink coffee in the 5 h prior to the study. The diet prior to the thermographic test was organized by the nutritionist in relation to the sports requirements of the athletes and the thermographic recommendations.

Image processing was performed by 2 blinded investigators using Flir IR research software following the previous analysis methods described by Cabizosu et al. [37]. For all measurements, the regions of interest (ROI) in the body were defined using conventional anatomical and scientific bibliography [39]. The thermographic measurements were carried out both in bipedal and seated positions, analyzing the quadricipital regions in the bipedal position and the patellar regions in the seated position. In order to be precise and reliable in the analysis of the anatomical regions, the anatomical limits of the patellar tendon were marked with a reflective sticker, placing a reflective sticker on the lower edge of the patella and another on the anterior tibial tuberosity. In this way, it was ensured that in the thermographic images, the anatomical limits of this structure had a different infrared emission gradient with respect to the skin temperature.

Phase 1: The athletes were acclimatized for a period of 20 min at 21–23 °C, a humidity of 40 (±0.8)%, and an atmospheric pressure of 1 ATM.

Phase 2 (baseline (B)): After the acclimatization period, the first thermographic acquisition was performed with the patients in a standing position for the anterior thigh region and in a seated position for the patellar region. In both cases, the athletes were located 1 m away from the thermograph, which was positioned on a fixed tripod.

Phase 3 (warm-up (W)): After the thermographic acquisition, the athletes completed a lower body ballistic warm-up exercise, recording again the temperatures of the regions studied.

Phase 4 (1st ELEC): Muscle peripheral properties of the participants’ right leg were assessed using a high-voltage (400 V) constant current research stimulator (DS7R, Digitimer, Hertfordshire, United Kingdom). Square-wave stimuli of 2 ms were applied to the quadriceps muscle using bipolar electrodes (9 × 5 cm). Electrodes were positioned to ensure that rectus femoris, vastus medialis, and vastus lateralis were stimulated. The position of the electrodes was marked on the skin with a permanent marker. Firstly, single twitches were applied with progressive increases of the current (10 mA) until the evoked twitch peak torque reached a plateau. The current was then increased by 20% to ensure that a supramaximal stimulation was applied. Consequently, this current intensity was used to apply two doublets (10 and 100 Hz).

Phase 5 (isokinetic): Participants performed two knee extension maximum voluntary isometric contractions (MVIC) at 90° knee flexion. They were asked to apply and maintain the maximal force for 5 s. Resting time between repetitions was 1 min. They had visual feedback of the torque-time series, and the signal was reviewed by the researcher to determine if there was a countermovement at the beginning of the contraction. After determining MVIC, participants performed a fatiguing test that consisted of maximal isokinetic repetitions of the knee extensors at 60°/s in concentric mode with a 90° range of motion (ROM). They were asked to apply maximum force in all repetitions and throughout the full ROM and to cease force production in knee flexion so that the hamstring muscles were not involved. The test ended when the maximal force achieved during the test decreased by 50% compared to the best repetition.

**Figure 1:**
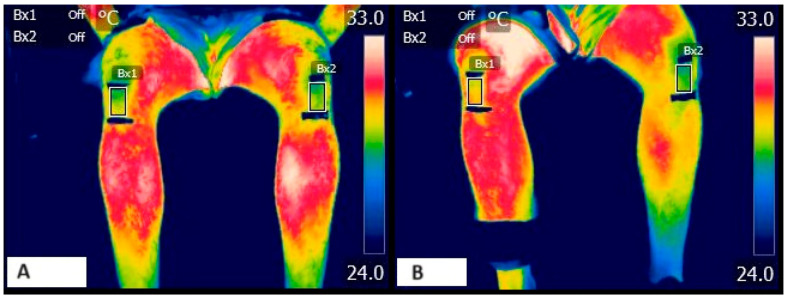
Patellar thermography during protocol. (**A**) Basal thermography and (**B**) final thermography.

Phase 6 (2nd ELEC): After the fatiguing task, two more doublets with the same intensity at 10 and 100 Hz were applied, as well as two knee extensors MVIC to assess the presence of neuromuscular fatigue (Figure 1).

#### 2.3.3. Anthropometry

A certified ISAK Level-1 researcher (FJMN) performed the anthropometric measurements. Height and body weight were measured using a digital scale with a stadiometer for clinical use (SECA 780; Vogel & Halke GmbH & Co., Hamburg, Germany). Skinfold thickness was measured using Holtain Skinfold Calipers (Holtain, Ltd., Crymych Pembrokeshire, UK) in accordance with the International Society for the Advancement of Kinanthropometry guidelines [40]. The percentage of body fat was determined using the Faulkner equation [41], while the percentage of muscle mass was calculated using the modified Matiegka equation [42]. The sum of the eight skinfolds (triceps, subscapular, bicep, iliac crest, supraspinal, abdominal, thigh, and calf) was also calculated.

#### 2.3.4. Statistical Analysis

IBM Social Sciences software (SPSS, v.21.0, Chicago, IL, USA) was used for statistical analysis. Data are presented as mean ± SD. Homogeneity and normality of the data were checked with the Levene and Shapiro–Wilk tests, respectively. For each ROI variable, a three-way repeated-measures ANOVA with time factor (BA vs. CAL vs. 1st ELEC vs. ISO vs. 2nd ELEC), sex factor (men vs. female), and side factor (right (R) and left (L)) were performed. Tukey’s post hoc analysis was carried out if significance was found in the ANOVA models. Partial eta squared (ηp2) was also calculated as the effect size for the interaction of all variables in the ANOVA analysis. Partial eta square thresholds were used as follows: <0.01, irrelevant; ≥0.01, small; ≥0.059, moderate; ≥0.138, large [43,44]. The different correlations between the parameters were evaluated using Pearson’s or Spearman’s correlation (r). Significance level was set at *p* ≤ 0.05.

## 3. Results

Assessing skin surface temperature changes by thermography can help to understand certain physiological processes that occur during exercise. Therefore, it is important to be able to quantify these changes in order to establish control criteria.

**Figure 2:**
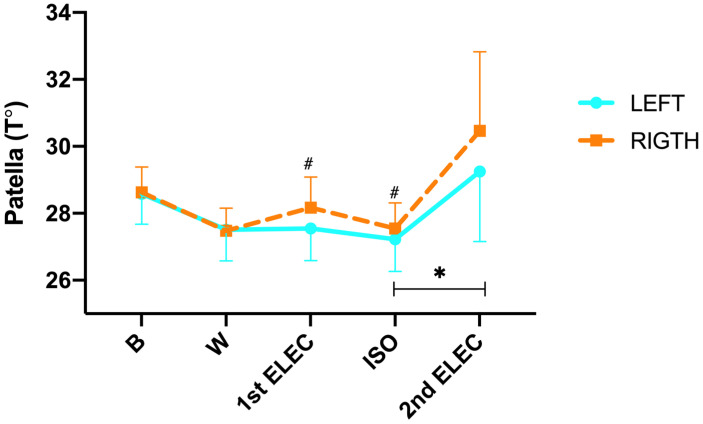
Patellar skin surface temperature changes were measured by infrared thermography during the exercise protocol. B = rest; W = warm-up; 1st ELEC = first electrostimulation; ISO = isokinetic and 2nd ELEC = second electrostimulation. * = *p* ≤ 0.05 between ISO and 2nd ELEC on the left leg. # = *p* ≤ 0.05 between both sides.

In relation to temperature changes in the patella (Figure 2), we found a significant difference in the time × side interaction (*p* ≤ 0.001; η^2^p = 0.366). In addition, during the protocol (intra-group analysis), post hoc Tukey’s found no significant changes from B to CAL (L: −1.1 °C; *p* = 0.408 and R: −0.9 °C; *p* = 0.536), from CAL to 1st ELEC (L: 0.04 °C; *p* = 1.000 and R: 0.46 °C; *p* = 0.966), and from 1st ELEC to ISO (L: −0.33 °C; *p* = 1.000 and R: −0.27 °C; *p* = 1.000), but, we did find a significant increase from ISO to 2nd ELEC in the left patellar (L: 1.7 °C; *p* = 0.007 and R: 2.6 °C; *p* = 0.256). On the other hand, post hoc Tukey’s showed significant differences in 1st ELEC (*p* = 0.016) and ISO (*p* = 0.003) between both sides. In addition, a significant positive correlation was found in the 2nd ELEC (r = 0.975; *p* ≤ 0.001) between the right and left sides. Moreover, a significant positive correlation was found in 1st ELEC (r = 0.824; *p* ≤ 0.001), ISO (r = 0.786; *p* ≤ 0.001), and 2nd ELEC (r = 1.000; *p* ≤ 0.001) between the right and left side.

**Figure 3:**
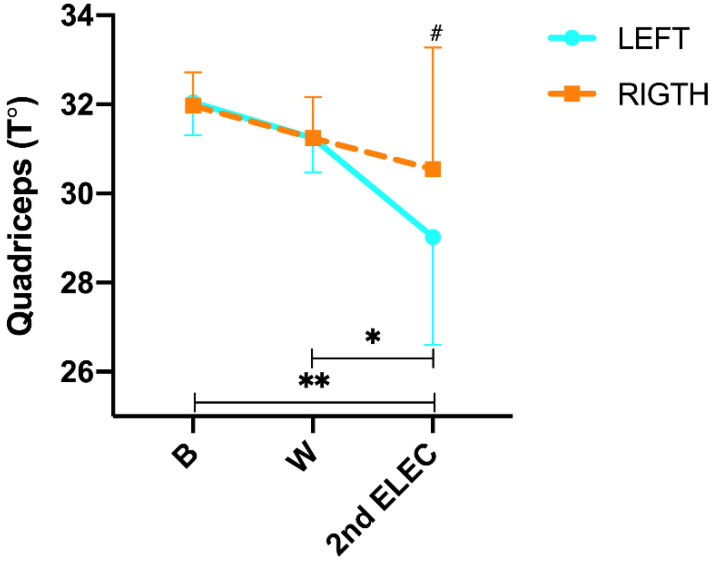
Quadriceps skin surface temperature changes were measured by infrared thermography during the exercise protocol. B = rest; W = warm-up; 1st ELEC = first electrostimulation; ISO = isokinetic and 2nd ELEC = second electrostimulation. * = *p* ≤ 0.05 between W and 2nd ELEC in the left leg. ** = *p* ≤ 0.05 between B and 2nd ELEC in both legs. # = *p* ≤ 0.05 between both sides.

Regarding quadriceps temperature changes (Figure 3), we found a significant difference in the time × side interaction (*p* ≤ 0.001; η^2^p = 0.786). In addition, during the protocol (intra-group analysis) post hoc Tukey´s found no significant changes from B to CAL (L: −1.6 °C; *p* = 0.422 and R: −1.6 °C; *p* = 0.347), but we did find a significant decrease in the T° from CAL to 2nd ELEC in QUA left (L: −2.2 °C; *p* = 0.005 and R: −0.8 °C; *p* = 0.684) and from B to 2nd ELEC (L: −3.2 °C; *p* ≤ 0.001 and R: −1.7 °C; *p* = 0.018). On the other hand, post hoc Tukey’s showed significant differences in 2nd ELEC (*p* ≤ 0.001) between both sides. In addition, a significant positive correlation was found in the 2nd ELEC (r = 0.975; *p* ≤ 0.001) between the right and left sides.

## 4. Discussion

The aim of this study was to observe and describe the bilateral skin temperature variation in national-level sprinters, in the anterior thigh region and patellar tendon, after the application of a unilateral muscle fatigue protocol carried out with electrostimulation and isokinetic knee extension exercises. The main findings found in this study generally show a decrease in temperature in muscular and tendon regions, although it should be noted that the tendon portion does not follow a homogeneous pattern of thermal regulation.

In the anterior thigh region, a progressive decrease in temperature was observed bilaterally as muscle fatigue increased, becoming more evident and statistically significant only in the left thigh at the end of the protocol, −3.2 °C; *p* < 0.001. These data confirm the results previously described by [24,45,46], which observed a reduction in temperature from baseline to the end of the muscle fatigue protocols performed. However, these findings contrast with those obtained by other authors, since, in these cases, an increase in temperature after fatigue in the most stressed region was observed [33,47,48]. Exercise physiology foresees that, during muscle contraction, due to the metabolic response of the structures involved, thermoregulatory and vascular changes are generated from the least stressed regions to the most stressed regions [49,50]. This process of blood redistribution is reflected in a variation in skin temperature, due to superficial vasoconstriction, as opposed to deep vasodilatation, regulated by cardiac output and arterial pressure according to oxygen demand at a deeper level [51,52].

According to some authors [53,54], the result of a reduction in circulation at the superficial level to favor an increase at the deep level, from the thermographic view, translates into a decrease in skin temperature, while for others [55,56,57], the result of deep overheating produced by muscle contraction produces an increase in skin temperature, affirming that there is a transmission effect of this heat at the superficial level, from the depth, which generates a release of heat by dissipation at the end of the exercise. In line with these authors, the results obtained in this study would be evidence that, on the one hand, thermoregulation is a global and not a local process. So, the decrease in temperature is observed bilaterally, and on the other hand, that the lower thermal decrease of the stressed limb at the end of the left protocol −3.2 °C and right, −1.7 °C, could be the result of unilateral overheating produced by muscle contraction.

The variation of the contralateral temperature could also be justified by an aspect that has been widely studied from the physiological point of view, but not thermographically, which is the crossover effect [58,59]. It is known that, due to the activation of commissural interneurons at the spinal level, it is possible to observe an activation of certain physiological and functional parameters in the contralateral limb, so that the temperature variation could represent a deep activation of the unsolicited system, not only from the point of view of blood flow redirection, but also of nervous activation [60]. Since the physiological processes that generate this response are not conclusively known, different authors have approached this phenomenon advancing several hypotheses. A possible explanation could be represented by the overflow of the neuronal electrical impulse that would generate contralateral activations or possible neuromuscular adaptations that would influence the contralateral side since improvements in the strength of the untrained side have been observed in unilateral training [61]. However, it should be noted that, from a thermographic point of view, a consensus has not yet been reached regarding the cross-over effect, since, on the one hand, as Dindorf et al. [49] observed, there is a statistical decrease in the temperature of the non-exercised limb at the end of the unilateral effort in different anatomical regions area: 1 *p* < 0.001; area 3 *p* < 0.001. On the other hand, Escamilla et al. [49] described an increase in temperature of up to +1°C after the end of the muscle fatigue protocol in poorly trained and highly trained individuals.

In this regard, it should be noted that the results obtained in this work were measured immediately after exercise, as were the results obtained by Dindorf et al. [62], while Escamilla et al. [58] performed the measurements 30 and 60 min after the end of the protocol. Based on previous studies, we then speculate on a model that predicts a progressive bilateral cooling process of skin temperature which will occur as deep energy expenditure increases. This decrease will be significant and progressive until it reaches a peak, at which point it will tend to stabilize until the end of the test. At the end of the test, we can observe in the following 10–15 min a process of return to the superficial thermal normality with progressive warming of the temperature with respect to the final phase of the effort [47,63,64] until an increase in basal temperature in the following 24–48 h due to systemic inflation related to the muscle repair process [65,66,67]. In order to confirm this model, it would be interesting to continuously record the variation of skin temperature for 48 h after a muscular fatigue protocol to confirm if and how long the thermal reorganization process lasts after exercise.

In relation to the tendon our, results show a first phase of thermoregulatory behavior similar to the muscular system with a decrease from B to W in the left tendon of −1.1 °C and on the right of −0.9 °C. However, this decrease is followed by an increase in temperature after the first electrotherapy, followed by a second decrease in temperature after the isokinetic, and finally followed by a final overheating left (+1.7 °C) and right (2.6 °C). Since the patellar tendon is a much more superficial structure with respect to the muscular bellies of the thigh, and since fat at this level is much more present than at the tendon level, our results could be evidence of the thermoregulatory model proposed above since, in a first phase of active heating, the blood would be directed towards the depth to meet the physiological demands of the tissue, generating a thermal decrease at the cutaneous level. In the second phase, after the 1st electro, we would be observing this superficial overheating since, in this phase, the protocol foresees the absence of motor activity in relation to the tendon, which becomes again a thermal decrease after the motor effort performed in the isokinetic. Immediately after the isokinetic, there is a cessation of motor activity at the tendon level, so the overheating observed in this last phase is the result of the heat generated and transmitted with the isokinetic. In this sense, our results represent an exclusivity since, to our knowledge, there are no known previous studies or reference values in professional runners that evaluate the thermoregulatory response in tendons after protocols that alternate isokinetic quadriceps exercises to electrostimulation under normal environmental conditions.

Although no similar previous studies are known that could justify the thermal challenge obtained in this work, the temperature increase observed in the second phase could be justified with the results proposed by other authors, since it is known that the responses to load, as in the muscular system, include mechanical and metabolic-circulatory variations at the tendon level [68]. According to some authors, even with short-duration exercises, changes in tendon microcirculation are generated, increasing the total hemoglobin values and intra-structural oxygen saturation as physiological stress increases [69]. From a thermographic point of view, this could translate into a thermal increase due to the increase in vascular demand generated after the initial heating process. However, it should be noted that this theory could contrast with the results obtained by other authors who correlated a thermal increase by thermographic imaging in relation to a process of tendon vascular restriction by echo-Doppler in patients with patellar tendinitis [17]. An increased thermal state in tendon pathology could represent the first state of autonomous tissue repair, as proposed in muscle structures [65]. In fact, as in the muscular system, this could be due to a previous thermal decrease as a result of overexertion. If we consider that the increase in vascularization, in non-pathological processes, coincides with a decrease in tendon stiffness and a greater elongation of the tendons, this could be due to a previous thermal decrease as a result of overexertion [70], and that in pathological processes an intra-tendinous vascular resistance is generated which results in an increase in stiffness and a decrease in its extensibility, it would be of extreme interest for future research, to observe by means of clinical thermography, the influence of different loads at the tendon level to relate them successively with recovery processes of the pathological state.

According to these results, thermography could be a useful tool to determine the metabolic and functional response obtained after a short-duration, high-intensity physiological muscle and tendon stress protocol in national-level runners. However, it should be considered that the small sample used in this study could represent a limiting factor. Future studies are needed in which the importance of the crossover effect on thermoregulatory processes is also analyzed, as it could influence the response to short-duration exercise. This would help to clarify differences in functional activation levels in relation to skin surface temperature that are not yet entirely clear. The observation of the metabolic response in all phases of exercise could be interesting in predicting the athlete’s response to high-intensity, short-duration work.

## 5. Conclusions

Our results confirmed that muscle exposure to high-intensity stress can generate significant changes in temperature patterns in the lower limbs, both in the muscle and tendon portions. In addition, we can observe that muscle activation during exercise affects both the homolateral and contralateral sides and is reflected in a change in skin temperature response. In addition, according to our results, the thermoregulatory effects at the tendon level appear much faster than at the muscle level, which could be of extreme interest not only to assess workloads in athletes but also for future research focused on the recovery and rehabilitation of the tendon system. Future research could be conducted to evaluate the predictive value of neuromuscular fatigue in the patellar tendon and quadriceps after exercise in order to optimize post-exercise recovery strategies. This study has also shown that, due to its easy handling and transport, thermography can be a good tool to follow and monitor instantaneously and, in situ, the muscle and tendon metabolic response in professional runners.

## 6. Limitations of the Study

One of the main limitations of this study was the small number of participants and the lack of similar research with which to compare results. However, although there is currently great controversy regarding thermoregulatory responses and the consequent cutaneous response in relation to muscle and tendon stress, our study may be of great use for future research in this field. Another limitation of the study could be represented by the type of thermograph used. In this regard, it should be noted that this tool is an industrial device not specifically manufactured for research with human beings. It is pertinent to point out that there is no updated registration by the Food and Drug Administration (FDA) or CE mark that classifies it as a medical device for research, although it has been validated in different studies as a diagnostic tool in humans.

## Data Availability

The data is available contacting with the corresponding author by email.
